# Factors that determine thione(thiol)–disulfide interconversion in a bis(thiosemicarbazone) copper(ii) complex†

**DOI:** 10.1039/c9ra01115c

**Published:** 2019-03-19

**Authors:** Haewon Jeong, Yeji Kang, Jin Kim, Byung-Kwon Kim, Seungwoo Hong

**Affiliations:** Department of Chemistry, Sookmyung Women's University Seoul 04310 Korea kimbk@sookmyung.ac.kr hsw@sm.ac.kr; Western Seoul Centre, Korea Basic Science Institute Seoul 03759 Republic of Korea

## Abstract

Solvent-, acidity-, and redox-responsive thione(thiol)–disulfide interconversion were achieved by a dinuclear copper(ii) complex bearing a bis(thiosemicarbazone) (bTSC) ligand. The role of copper(ii) ion coordination was rationalized by parallel comparison with a bare bTSC ligand and zinc(ii) bTSC complexes under identical reaction conditions.

## Abstract 2

Solvent-, acidity-, and redox-responsive thione(thiol)–disulfide interconversion were achieved by a dinuclear copper(ii) complex bearing a bis(thiosemicarbazone) (bTSC) ligand.

## Graphical abstract

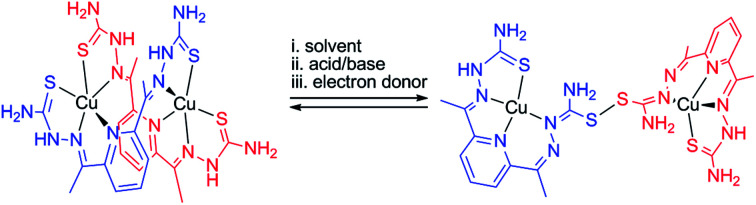

The chalcogen elements such as oxygen, sulfur, and selenium are of substantial biological and technological relevance due to their tremendous potential for industrial and biomimetic applications. In biological systems, thione(thiol)–disulfide exchange reactions play a critical role in a myriad of enzymatic machinery for cellular functions including redox activity, oxidative protein folding, DNA repair/expression, and apoptosis.^1^ In particular, S-based amino acids such as cysteine and methionine provide a rich redox chemistry by spanning fractional oxidation states up to ten (*e.g.*, from +6 to −2).^2^ Disulfides are also employed as key compounds in many chemical processes such as a protecting group in organic synthesis, vulcanizing agents for rubber and a sulphenylating agent for enolates and anions.^3^ Therefore, understanding factors that control the reversible S–S bond cleavage and formation has come to be a prominent challenge in diverse research fields.

Although extensive mechanistic studies on thiol–disulfide exchange have been established for use in dynamic combinatorial chemistry,^4^ thione–disulfide exchange has been less explored. In this context, the use of thiosemicarbazones (TSCs) that contain a carbothioamide group would be an attractive mechanistic blueprint to provide information regarding thione–disulfide interconversion.

In this work, we explored bis(thiosemicarbazone) (bTSC) as a chelating ligand of copper(ii) and zinc(ii) ions to probe the thione–disulfide interconversion mechanism. These complexes are observed to control the scission and formation of the S–S bond, promoted by (i) solvent system, (ii) acid–base treatments, and (iii) redox processes with the help of the copper(ii) ion coordination (Scheme 1).

**Scheme 1:**
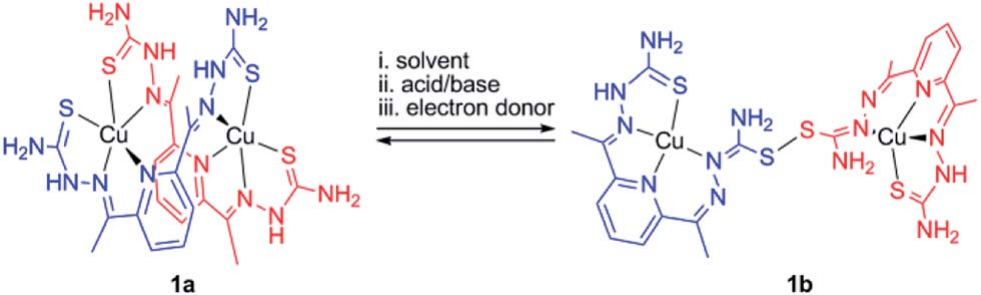
Plausible outline for thione–disulfide interconversion by a dinuclear copper(ii) bis(thiosemicarbazone) complex.

A green dimeric copper(ii) complex bearing the bTSC ligand (H_2_N_3_S_2_), [Cu(H_2_N_3_S_2_)_2_](CH_3_CN)(CF_3_SO_3_)_4_ (1a), was prepared by following modified literature procedure,^5^ and characterized by various spectroscopic methods such as UV-vis and electron paramagnetic resonance (EPR) spectroscopies, electrospray ionization mass spectrometry (ESI MS), and X-ray crystallography (details can be found in ESI†). UV-vis spectrum of 1a in CH_3_CN exhibited an intense charge transfer band at 281 nm (*ε* = 5.5 × 10^4^ M^−1^ cm^−1^) and two broad d–d transition at 580 nm (*ε* = 450 M^−1^ cm^−1^) and 690 nm (*ε* = 390 M^−1^ cm^−1^) (Fig. S1a, ESI†). An ESI MS spectrum of 1a exhibited one prominent peak at *m*/*z* of 890.9, whose mass and isotopic distribution patterns correspond to {[Cu_2_(H_2_N_3_S_2_)_2_](CF_3_SO_3_)}^+^ (calculated *m*/*z* of 891.0) (Fig. S1b, ESI†). An EPR spectrum of 1a clearly showed the presence of a copper(ii) ion with *g* value of 2.02 (Fig. S2a, ESI†). The X-ray crystal structure of 1a revealed a dinuclear copper complex with helical structure due to two rotations around the symmetrical C–C bond adjacent to the pyridine ring (Fig. 1, Table S1 and S4, ESI†). It has been well-documented that the rotational flexibility of bTSC ligand afforded different configurations with multiple binding modes.^6^ This configuration mode provokes that each copper(ii) center is in a distorted square pyramidal N_3_S_2_ environment. The average C–S bond length of 1.711(4) Å in bTSC ligand probably retain a partial double bond character (*e.g.*, the C=S bond length of 1.672–1.700 Å in pyridine-2-(1*H*)-thione *vs.* the average C–S bond length of 1.8 Å in typical thiol group).^7^

**Fig. 1:**
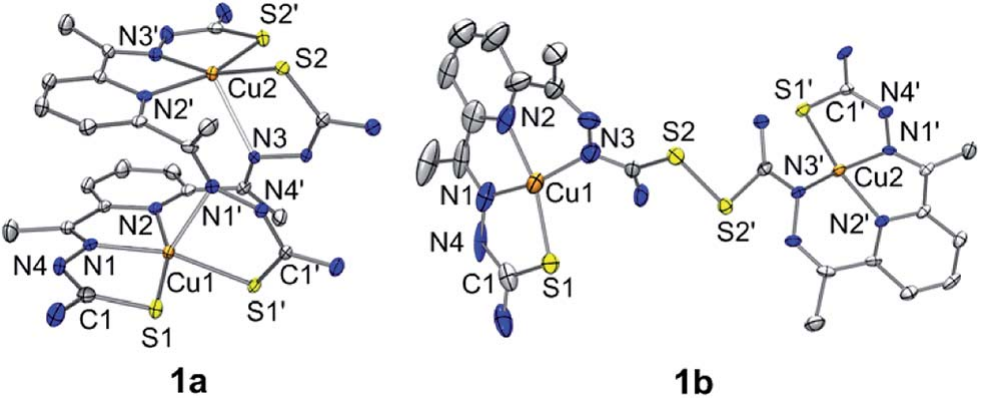
X-ray crystal structures of dinuclear copper(ii) bis(thiosemicarbazone) complexes with thermal ellipsoids showing 50% probability. Triflate ions, solvent molecules and hydrogen atoms are omitted for clarity.

When an isolated green solid 1a was dissolved in CH_3_OH, a brown colour solution was obtained. UV-vis spectrum of the new brown complex, denoted as 1b, displayed red-shifted electronic absorption band at 306 nm (*ε* = 5.8 × 10^4^ M^−1^ cm^−1^), and blue-shifted d–d bands 419 nm (*ε* = 1.1 × 10^4^ M^−1^ cm^−1^) and 540 nm (*ε* = 510 M^−1^ cm^−1^) (Fig. S1a, ESI†). An EPR spectrum of 1b clearly showed the presence of a copper(ii) ion with *g* value of 1.98 (Fig. S2b, ESI†). Titration experiment was performed by adding aliquot amount of CH_3_OH into the CH_3_CN-solution containing 1a; a clean conversion from 1a to 1b was monitored by UV-vis spectroscopy (Fig. S3, ESI†). Very interestingly, the molecular structure of [Cu(HN_3_S_2_)_2_](CH_3_OH)(CF_3_SO_3_)_4_ (1b) revealed that two bTSC ligands were bridged through a S–S bond and each copper(ii) center exhibited slightly distorted square planar geometry (Fig. 1, Table S1 and S4†). The S–S bond length of 2.069(3) Å is in a good agreement with reported disulfide bond length (Fig. 1 and Table S4†).^8^ Considering the elongation of C1–S2 bond length (*e.g.*, from 1.712(5) to 1.799(5) Å) along with the shortening of C1–N3 bond (*e.g.*, from 1.361(7) to 1.327(9) Å), the S–S bond formation occurred at the expense of double bond rearrangement from initial C=S bond to C=N bond. In addition, when the isolated 1b was dissolved in CH_3_CN, the spectroscopic feature was identical to 1a (Fig. S1†). In fact, CH_3_OH might act as a base when 1a was dissolved in CH_3_OH since CH_3_OH is amphoteric. The deprotonation of hydrazino moiety of thiosemicarbazone engendered thiolate anion *via* double bond rearrangement and the nucleophilic attack of thiolate anion on the sulfur atom of another thiosemicarbazone resulted in the disulfide bond formation.^4^ Therefore, a quantitative solvent-responsive thione–disulfide interconversion was evidenced by changing solvent system.

Intrigued by the S–S bond scission and formation within bTSC ligand system, we attempted to scrutinize other exogenous factors that directly affect the thione–disulfide interconversion reaction. Given the fact that the disulfide bond formation of 1a occurred along with the double bond rearrangement *via* a deprotonation of hydrazino moiety in bTSC ligand, the deprotonation of 1a could presumably be a possible synthetic route for the formation of disulfide bond. Indeed, it is well-established that in thione–thiol tautomerism,^9^ thione form prevails in neutral and acidic media while the equilibrium shifts toward thiol in alkaline medium. Taken together, thione containing ligands might be susceptible to undergo the disulfide bond formation unless they could shift the equilibrium toward thiol *via* proton transfer (*e.g.*, p*K*_a_ of thione ligands) and double bond rearrangement.

Addition of 2.2 equiv. of KOH to the reaction solution of 1a in CH_3_CN resulted in the immediate solution colour changes from green to brown, indicating the immediate conversion of 1a to 1b. (Fig. S4, ESI†). Titration experiment clearly suggested the 1 : 2 deprotonation of 1a (Fig. 2). Remarkably, further addition of 3.0 equiv. of HClO_4_ to the reaction solution containing 1b led to a quantitative recover of 1a (Fig. S4, ESI†). This interconversion cycle could be repeated several times. Thus, 1a and 1b can be readily interconverted through the acid–base chemistry in plausible consequence of thione–thiol equilibrium shift.

**Fig. 2:**
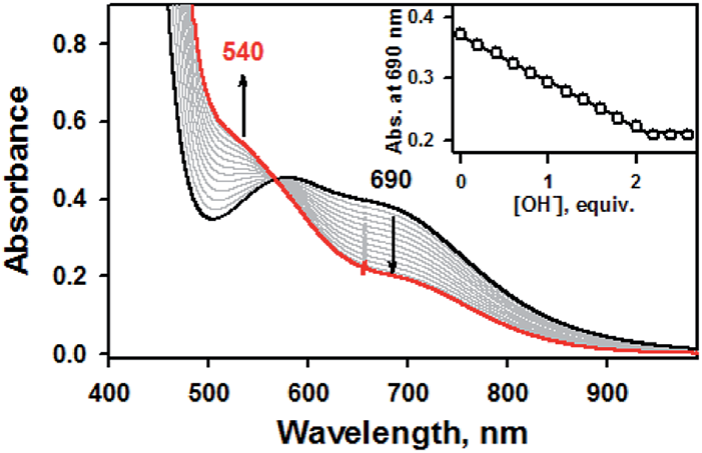
UV-vis spectral changes showing the formation of 1b (red line) and disappearance of 1a (1.0 mM, black line) upon addition of KOH to 1a in increment of 0.20 equiv. in CH_3_CN at 20 °C. Inset shows the plot of absorbance changes at 690 nm due to 1a (black dot) and against the equivalents of KOH added to 1a in CH_3_CN at 20 °C.

Furthermore, it has been well-documented that the redox processes are involved in the thiol–disulfide exchange in biological system.^10^ For instance, oxidative protein folding due to the transformation of glutathione (GSH) to glutathione disulfide (GSSG) has extensively examined by *in vivo* studies.^10^ To examine this redox-induced thiol(thione)–disulfide exchange, we examined the electrochemical properties of 1a and 1b. In electron transfer reaction, 1a disappeared with a first-order rate profile with a concomitant formation of 1b upon addition of ferrocene (Fc, *E*_ox_ = 0.37 V *vs.* SCE); a second-order rate constant (*k*_2_) of 6.3 M^−1^ s^−1^ was obtained at 10 °C (Fig. 3). This result indicated that one-electron reduction potential of 1a is between above 0.37 V *vs.* SCE. This was also confirmed by cyclic voltammetry, providing that the *E*_red_ of 1a is 0.52 V *vs.* SCE (Fig. S5, ESI†). Reasonably, the presence of electron source might facilitate the thiol formation over the thione within bTSC ligand. Taken together, by virtue of the copper(ii) ion coordination combined with three exogenous factors including solvent, acid/base, and electron donor, thione was converted to thiol prior to the S–S bond formation. Indeed, it has been reported that chalcogenone ligands could be converted to the corresponding dichalcogenide ligands in the presence of copper(ii) ion.^11^ This phenomenon is greatly relevant to the co-existence of copper(ii) ion and GSH in the biological system since it has been proposed that the tight control of the copper(ii)–GSH complex and the depletion of GSH level could prevent the copper-dependent DNA damage and certain diseases such as Wilson's disease and rheumatoid arthritis.^12^

**Fig. 3:**
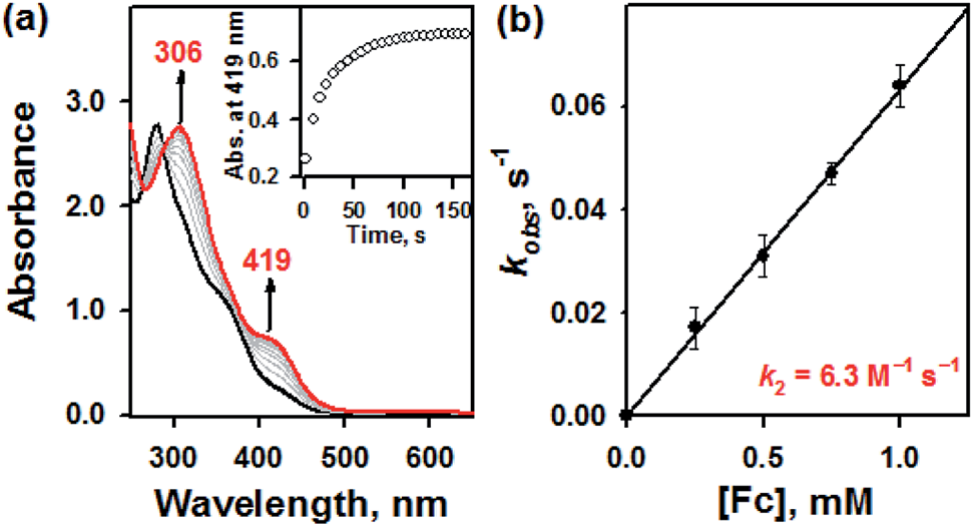
(a) UV-vis spectra of the reaction of 1a (0.050 mM) with ferrocene (0.25 mM) in CH_3_CN at 10 °C. (b) Plot of pseudo-first-order rate constants (*k*_obs_) against the concentrations of ferrocene to determine the *k*_2_ value for 1a in CH_3_CN at 10 °C.

To scrutinize the role of copper, we explored the thione–disulfide exchange reactions by using a bare bTSC ligand and a zinc(ii) complex bearing the bTSC ligand serving as a redox-inactive metal coordinating complex. When a bare bTSC ligand was dissolved in different solvents such as CH_3_CN and CH_3_OH, we did not observe any distinct features (Fig. S6a, ESI†). Furthermore. the thione–disulfide exchange did not upon neither direct treatment of an acid/base or an electron donor into a solution containing the bTSC ligand (Fig. S6b and S6c, ESI†). Therefore, the sole bTSC ligand without the copper(ii) ion coordination could not the reversible thione–disulfide interconversion.

The zinc(ii) complexes bearing the bTSC ligand were prepared in both CH_3_CN and CH_3_OH, denoted as 2a and 2b, respectively (details can be found in ESI†). UV-vis spectra of 2a and 2b displayed characteristic bands at 272 nm (*ε* = 2.7 × 10^4^ M^−1^ cm^−1^) in CH_3_CN and 278 nm (*ε* = 2.7 × 10^4^ M^−1^ cm^−1^) in CH_3_OH, respectively (Fig. S7, ESI†). The solid-state X-ray structures of 2a and 2b revealed completely different geometries; while 2a presented a sulfur-bridged dinuclear zinc(ii) complex in a distorted pentagonal geometry, 2b was pentagonal bipyramidal geometry (Fig. 4, Table S3 and S5 ESI†). When an isolated solid 2a was dissolved in CH_3_OH, the spectroscopic feature was identical to 2b, and *vice versa* (Fig. S7, ESI†). Thus, a solvent-responsive thione–disulfide exchange reaction did not occur when a zinc(ii) ion was coordinated to bTSC ligand.

**Fig. 4:**
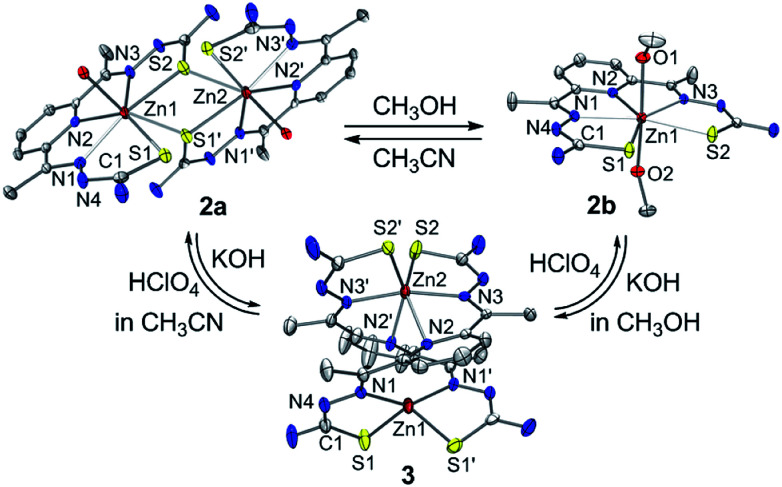
X-ray crystal structures of solvent- and acidity-dependent zinc(ii) bis(thiosemicarbazone) complexes with thermal ellipsoids showing 50% probability.

To aid comparison, acidity-, and redox-responsive thione–disulfide exchange reaction in the identical reaction conditions were also reassessed. Addition of 2.2 equiv. of KOH to either yellow solutions of 2a or 2b afforded an identical deep yellow complex. The deprotonated complex, denoted as 3, displayed characteristic absorption bands at 336 nm (*ε* = 3.5 × 10^4^ M^−1^ cm^−1^) and a shoulder at 400 nm (*ε* = 1.5 × 10^4^ M^−1^ cm^−1^) (Fig. S8, ESI†). Titration experiment clearly suggested the 1 : 2 deprotonation of 2a (Fig. 5). While ^1^H NMR spectrum of 2a showed a clear signal at 10.3 ppm, which is indicative of NH at hydrazino moiety within bTSC ligand, 3 did not show any NH signal indicating the deprotonation at hydrazino moiety (Fig. S9, ESI†). Furthermore, the molecular structure of 3 revealed a dimeric complex with the elongation of C–S bonds length from 1.695(4) to 1.740(3) Å due to the double bond rearrangement (Fig. 4, Table S3 and S5, ESI†); this is consistent with the dimeric zinc(ii) complex having the C–S single bond character (*e.g.*, bis(thiosemicarbazonato) ligand). Interestingly, upon addition of 3.0 equiv. of HClO_4_, 3 rapidly reverts back to 2a in CH_3_CN or to 2b in CH_3_OH, suggesting that (i) 3 and 2 can be readily interconverted through the acid–base reaction and (ii) the acid–base equilibria are also solvent dependent (Fig. 4 and S8, ESI†). We further attempted to introduce an electron donor into the solution of 2a and 3 in order to promote S–S bond formation, however, thione–disulfide exchange did not take place (Fig. S10, ESI†). On the basis of these series of control experiments by using the bTSC ligand and a zinc(ii) complex bearing bTSC ligand, a copper(ii) ion coordination might be preceded by other exogenous factors such as solvent, acidity of media and redox process.

**Fig. 5:**
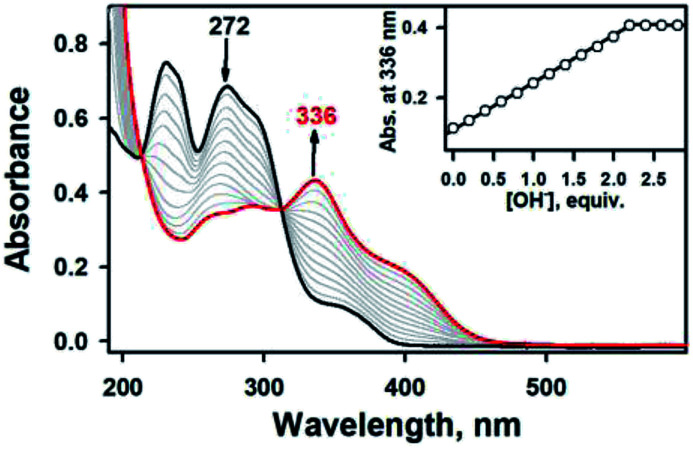
UV-vis spectral changes showing the formation of 3 (red line) and disappearance of 2a (0.025 mM, black line) upon addition of KOH to 2a in increment of 0.20 equiv. in CH_3_CN at 20 °C. Inset shows the plot of absorbance changes at 336 nm due to 3 (black dot) and against the equivalents of KOH added to 2a in CH_3_CN at 20 °C.

To summarize, we have successfully synthesized and characterized copper(ii) and zinc(ii) complexes bearing bTSC ligand. Given the presence of thione group on the bTSC ligand, this synthetic approach facilitates direct investigations on the thione–disulfide interconversion among bare bTSC ligand, copper(ii), and zinc(ii) complexes bearing bTSC ligand as a function of exogenous factors such as solvent, acid/base, and electron donor. As demonstrated above, thione–disulfide interconversion can be modulated by several exogenous factors accompanied by the copper(ii) ion coordination.

## Conflicts of interest

There are no conflicts to declare.

## Supplementary Material

RA-009-C9RA01115C-s001

RA-009-C9RA01115C-s002
