# Supplementary material for: Factors that determine thione(thiol)–disulfide interconversion in a bis(thiosemicarbazone) copper(ii) complex
Source: RSC Adv. 2019 Mar 19;9(16):9049–52. doi: 10.1039/c9ra01115c (PMC9062045; doi:10.1039/c9ra01115c)
Supplement: RA-009-C9RA01115C-s001 [file RA-009-C9RA01115C-s001.pdf]

Electronic Supplementary Material (ESI) for RSC Advances.
This journal is © The Royal Society of Chemistry 2019

Supporting Information

Factors that determine thione(thiol)-disulfide interconversion in bis(thiosemicarbazone) copper(II) complex

Haewon Jeong,[a] Yeji Kang,[a] Jin Kim,[b] Byung-Kwon Kim*[a] and Seungwoo Hong*[a]

[a] *Department of Chemistry, Sookmyung Women's University, Seoul 04310, Korea.*

[b] *Western Seoul Centre, Korea Basic Science Institute, Seoul 03759, Republic of Korea*

E-mail: kimbk@sookmyung.ac.kr; hsw@sm.ac.kr

Experimental Section

Materials and Instrumentation. Commercially available chemicals were used without further purification unless otherwise indicated. Solvents were dried according to published procedures and distilled under Ar prior to use.[S1] Bis(thiosemicarbazone) (bTSC) ligand, copper(II) and zinc(II) complexes bearing bTSC ligand were synthesized according to the literature.[S2] UV-vis spectra were recorded on a Hewlett Packard Agilent Cary 8454 UV-visible spectrophotometer equipped with a T2/sport temperature controlled cuvette holder. Electrospray ionization mass spectra (ESI MS) were collected on a Thermo Finnigan (San Jose, CA, USA) LTQ^{TM} XL ion trap instrument, by infusing samples directly into the source at 5.0 μL/min using a syringe pump. The spray voltage was set at 4.7 kV and the capillary temperature at 120 ^{o}C. ^{1}H NMR spectra were measured with a Bruker model digital AVANCE III 400 MHz FT-NMR spectrometer. Electrochemical measurements were performed on a CHI617B electrochemical analyzer (CH Instruments, Inc.) in CH_3CN containing 0.10 M Bu_4NPF_6 ($TBAPF_6$) as a supporting electrolyte at 25 ^{o}C. X-band EPR spectra were recorded at 77 K using an X-band Bruker EMX-plus spectrometer equipped with a dual mode cavity (ER 4116DM). Low temperatures were achieved and controlled with an Oxford Instruments ESR900 liquid He quartz cryostat with an Oxford Instruments ITC503 temperature and gas flow controller. A conventional three-electrode cell was used with a glassy carbon working electrode (surface area of 0.030 cm^{2}), a platinum wire as a counter electrode and an Ag/Ag^{+} electrode as a reference electrode. The glassy carbon working electrode was routinely polished with BAS polishing alumina suspension and rinsed with acetone and acetonitrile before use. The measured potentials were recorded with respect to an Ag/Ag^{+} (0.010 M) reference electrode. All potentials (vs Ag/Ag^{+}) were converted to values vs SCE by adding 0.29 V.[S3]

Synthesis and Characterization of Dinuclear Copper(II) Complexes, 1a and 1b. The dinuclear copper(II) complexes bearing bTSC ligand, $[Cu^{II}(H_2bTSC)_2](CH_3CN)(CF_3SO_3)_4$ (**1a**), and $[Cu^{II}(H_2bTSC)_2](CH_3OH)(CF_3SO_3)_4$ (**1b**) were prepared by reacting bTSC ligand and $Cu(CF_3SO_3)_2$ in CH_3CN and CH_3OH, respectively. UV-vis spectra of the copper(II) complexes exhibited distinct spectroscopic features at 281 nm for **1a** and 306 nm for **1b**. Both

ESI MS spectra of **1a** and **1b** displayed a prominent peak at *m/z* of 890.9, whose mass and isotopic distribution correspond to $\{[Cu_2(H_2bTSC)_2](CF_3SO_3)\}^+$ (calculated *m/z* of 891.0).

Synthesis and Characterization of Mono- and Dinuclear Zinc(II) Complexes, 2a, 2b, and 3. The mono- and dinuclear zinc(II) complexes bearing bTSC ligand, $[Zn_2^{II}(H_2bTSC)_2(H_2O)_2](CF_3SO_3)_4$ (**2a**), and $[Zn^{II}(H_2bTSC)(H_2O)](CF_3SO_3)_2$ (**2b**) were prepared by reacting bTSC ligand and $Zn(CF_3SO_3)_2$ in CH_3CN and CH_3OH, respectively. $[Zn_2^{II}(bTSC)_2]$ (**3**) was synthesized by adding 2.2 equiv of KOH into a solution containing **2a** in CH_3CN at 20 °C. ^1H NMR spectra of diamagnetic **2a** and **3** were taken at 20 °C in deutrated DMSO. A NH peak at 10.3 ppm assignable to a hydrazino moiety in **2a** was disappeared in **3** by the deprotonation of NH hydrazino group.

X-Ray Structural Analysis. Single crystals of dimeric copper(II) complexes **1a** and **1b** suitable for X-ray crystallographic analyses were obtained by slow diffusion of Et_2O into a CH_3CN solution of **1a** and a CH_3OH solution of **1b**, resepctively. Single crystals of mono- and dimeric zinc(II) complexes, **2a**, **2b** and **3** were obtained by slow diffusion of Et_2O into a CH_3CN solution of **2a** and **3** and a CH_3OH solution of **2b**, resepctively. These crystals were taken from the solutions by a nylon loop (Hampton Research Co.) on a hand made cooper plate and mounted on a goniometer head in a N_2 cryostream. The diffraction data for **1a**, **1b**, **2a**, **2b**, and **3** were collected at 120 K on a Bruker SMART AXS diffractometer equipped with a monochromator in the Mo *K*α (λ = 0.71073 Å) incident beam. Cell parameters were determined and refined by the SMART program.[S4] The CCD data were integrated and scaled using the Bruker-SAINT software package.[S5] An empirical absorption correction was applied using the SADABS program.[S6] The structures were solved by direct methods, and all non-hydrogen atoms were subjected to anisotropic refinement by full-matrix least squares on F^2 by using SHELXTL Ver. 6.14.[S7] Unless otherwise noted, hydrogen atoms were placed at their geometrically calculated positions and refined riding on the corresponding carbon, nitrogen or oxygen atoms with isotropic thermal parameters. For **1a**, **2a**, **2b**, and **3**, all N–H and O–H hydrogen atoms were located in the Fourier difference map and their positions were freely refined. The crystallographic data and selected bond distances and angles are listed in Tables S1-S5, respectively. Full crystallographic details can be obtained free of charge from

the Cambridge Crystallographic Data Centre via www.ccdc.cam.ac.uk/data_request/cif (CCDC 1895981-1895985 for **1a**-**3**)..

Reactivity Studies. Reactions were run in a 1-cm UV cuvette and followed by monitoring UV-vis spectral changes of reaction solutions. The thione-disulfide conversion from **1a** to **1b** was titrated by the successive addition of KOH (0.20 – 2.2 equiv) to the solution of **1a** (1.0 mM) in CH_3CN at 20 °C. The titration data were obtained by monitoring UV-vis spectral changes at 690 nm due to **1b**. Knowing the extinction coefficients of **1b** (690 nm; ε = 390 M^{-1} cm^{-1}), quantitative yields was determined by comparing the extinction coefficients obtained after the completion of disulfide bond formation. Titration experiments for the acid/base chemistry of mono- and dimeric zinc(II) complexes were conducted upon successive introduction of KOH (0.20 – 2.2 equiv) to the solution of **2a** (0.025 mM) in CH_3CN at 20 °C. Overall reaction rate constant and second-order rate constant (k_2) of the reaction between **1a** and ferrocene were determined under pseudo-first-order conditions by fitting the changes in absorbance at 419 nm due to **1b** and compared in order to determine the rate-determining step of the conversion from thione to disulfide. The kinetic experiments were run at least in triplicate, and the data reported represent the average of these reactions.

References

[S1] W. L. F. Armarego and C. L. L. Chai, *Purification of Laboratory Chemicals*, 6th ed.; Pergamon Press: Oxford, 2009.

[S2] A. I. Mantesanz, I. Cuadrado, C. Pastor and P. Souza, *Z. Anorg. Allg. Chem.*, 2005, **631**, 780.

[S3] C. K. Mann and K. K. Barnes, in *Electrochemical Reactions in Non-aqueous Systems*, Mercel Dekker, New York, 1970.

[S4] *SMART*, Data collection software; Bruker AXS Inc.: Madison, Wisconsin, USA, 2012.

[S5] *SAINT*, Data integration software; Bruker AXS, Inc.: Madison, Wisconsin, USA, 2012.

[S6] Sheldrick, G. M. *SADABS*, Program for absorption correction with the Bruker

SMART system; Universität Göttingen, Germany, 1996.

[S7] Sheldrick, G. M. *SHELXTL*, *Version 6.14*; Bruker AXS Inc.: Madison, Wisconsin, USA, 2003.

Table S1. Crystallographic Data and Refinements for **1a** and **1b**

	**1a**	**1b**
Empirical formula	$C_{32}H_{43}Cu_2F_{12}N_{15}O_{13}S_8$	$C_{28}H_{36}Cu_2F_{12}N_{14}O_{14}S_8$
Formula weight	1457.37	1404.27
Temperature (K)	120	120
Wavelength (Å)	0.71073	0.71073
Crystal system/space group	monoclinic, $C_{2/c}$	monoclinic, $P_{21/n}$
Unit cell dimensions		
a (Å)	30.369(9)	9.406(3)
b (Å)	11.838(4)	12.823(4)
c (Å)	36.838(11)	42.578(14)
α (°)	90	90
bβ (°)	107.677(4)	95.368(5)
γ (°)	90	90
Volume (Å^3)	12618(6)	5113(3)
Z	8	4
Calculated density (g/cm^{-3})	1.534	1.824
Absorption coefficient (mm^{-1})	1.036	1.276
Reflections collected	77806	29539
Absorption correction	multi-scan (T_{min} = 0.672, T_{max}= 0.745)	multi-scan (T_{min} = 0.680, T_{max}= 0.745)
Independent reflections	10996	8812
Goodness-of-fit on F^2	1.065	1.024
R [F^2 > 2sigma(F^2)]	0.0509	0.1148
wR^2	0.1211	0.3171

Table S2. Crystallographic Data and Refinements for **2a** and **2b**

	**2a**	**2b**
Empirical formula	$C_{15}H_{23}F_6N_7O_8S_4Zn$	$C_{26}H_{34}F_{12}N_{14}O_{14}S_8Zn_2$
Formula weight	737.01	1381.89
Temperature (K)	120	120
Wavelength (Å)	0.71073	0.71073
Crystal system/space group	monoclinic, $P_{21/C}$	monoclinic, $P_{21/n}$
Unit cell dimensions		
a (Å)	12.1424(17)	12.995(4)
b (Å)	15.372(2)	13.604(4)
c (Å)	14.688(2)	14.336(4)
α (°)	90	90
β (°)	90.086(2)	108.240(4)
γ (°)	90	90
Volume (Å^3)	2741.5(7)	2407.2(12)
Z	4	2
Calculated density (g/cm^{-3})	1.786	1.907
Absorption coefficient (mm^{-1})	1.296	1.466
Reflections collected	38665	29353
Absorption correction	multi-scan (T_{min} = 0.682, T_{max}= 0.745)	multi-scan (T_{min} = 0.601, T_{max}= 0.745)
Independent reflections	5687	4251
Goodness-of-fit on F^2	1.066	1.082
R [F^2 > 2sigma(F^2)]	0.0246	0.0432
wR^2	0.0648	0.1191

Table S3. Crystallographic Data and Refinements for **3**

	**3**
Empirical formula	$C_{25}H_{38}N_{14}O_3S_4Zn_2$
Formula weight	841.67
Temperature (K)	120
Wavelength (Å)	0.71073
Crystal system/space group	monoclinic, $P_{21/c}$
Unit cell dimensions	
a (Å)	13.697(3)
b (Å)	14.759(3)
c (Å)	18.746(4)
α (°)	90
β (°)	109.166(3)
γ (°)	90
Volume (Å^3)	3579.5(14)
Z	4
Calculated density (g/cm^{-3})	1.562
Absorption coefficient (mm^{-1})	1.624
Reflections collected	43448
Absorption correction	multi-scan (T_{min} = 0.658, T_{max}= 0.745)
Independent reflections	6296
Goodness-of-fit on F^2	1.025
R [F^2 > 2sigma(F^2)]	0.0361
wR^2	0.0916

Table S4. Selected Bond Distances (Å) and Angles (°) for **1a** and **1b**

	**1a**	**1b**
Bond Distances (Å)		
Cu1-N1	1.985(3)	1.926(13)
Cu1-N2	2.110(3)	1.973(11)
Cu1-N3	-	1.970(12)
Cu1-N1’	2.329(3)	-
Cu1-S1	2.3121(13)	2.276(4)
Cu1-S1’	2.3048(12)	-
Cu2-N3	2.344(3)	-
Cu2-N1’	-	1.969(9)
Cu2-N2’	2.122(3)	1.988(8)
Cu2-N3’	1.986(3)	1.943(9)
Cu2-S2	2.3068(12)	-
Cu2-S1’	-	2.300(3)
Cu2-S2’	2.3293(12)	-
C1-N4	1.359(5)	1.389(19)
C1-S1	1.718(4)	1.717(13)
S2-S2’	-	2.069(4)
Bond Angles (°)		
N1-Cu1-N2	78.71(13)	82.5(6)
N1-Cu1-S1	84.34(11)	86.9(4)
N2-Cu1-N3	-	88.1(5)
N2-Cu1-N1’	104.70(12)	-
N1’-Cu1-S1’	80.78(8)	-
N3-Cu2-S2	80.37(8)	-
N3-Cu2-N2’	105.39(12)	-
N1’-Cu2-N2’	-	80.2(4)
N1’-Cu2-S1’	-	85.1(3)
N2’-Cu2-N3’	78.53(13)	90.7(4)
N3’-Cu2-S2’	83.64(10)	-

Table S5. Selected Bond Distances (Å) and Angles (°) for **2a**, **2b**, and **3**

	**2a**	**2b**	**3b**
Bond Distances (Å)			
Zn1-N1	2.3784(14)	2.285(4)	2.074(3)
Zn1-N2	2.2538(15)	2.196(3)	-
Zn1-N3	2.3688(14)	2.347(4)	-
Zn1-S1	2.6435(5)	2.4912(12)	2.3336(11)
Zn1-S2	2.5431(5)	2.7129(13)	-
Zn1-O1	2.0854(12)	2.120(3)	-
Zn1-N1’	-	-	2.059(3)
Zn1-S1’	-	-	2.3461(10)
Zn2-N2	-	-	2.539(3)
Zn2-N3	-	-	2.082(3)
Zn2-S2	-	-	2.4014(10)
Zn2-N2’	-	-	2.359(3)
Zn2-N3’	-	-	2.079(3)
Zn2-S2’	-	-	2.3834(10)
C1-N4	1.354(2)	1.344(5)	1.322(4)
C1-S1	1.6842(17)	1.696(5)	1.750(3)
Bond Angles (°)			
N1-Zn1-N2	68.59(5)	70.69(12)	-
N1-Zn1-S1	72.55(4)	75.88(9)	84.65(8)
N2-Zn1-N3	68.78(5)	68.59(6)	-
N3-Zn1-S2	73.72(4)	69.76(12)	-
N1’-Zn1-N1	-	-	147.36(11)
N1’-Zn1-S1’	-	-	84.73(8)
N2-Zn2-N3	-	-	102.75(10)
N3-Zn2-S2	-	-	83.84(8)
N2’-Zn2-N2	-	-	90.58(9)
N2’-Zn2-N3’	-	-	74.4(1)
N3’-Zn2-S2’	-	-	82.00(8)

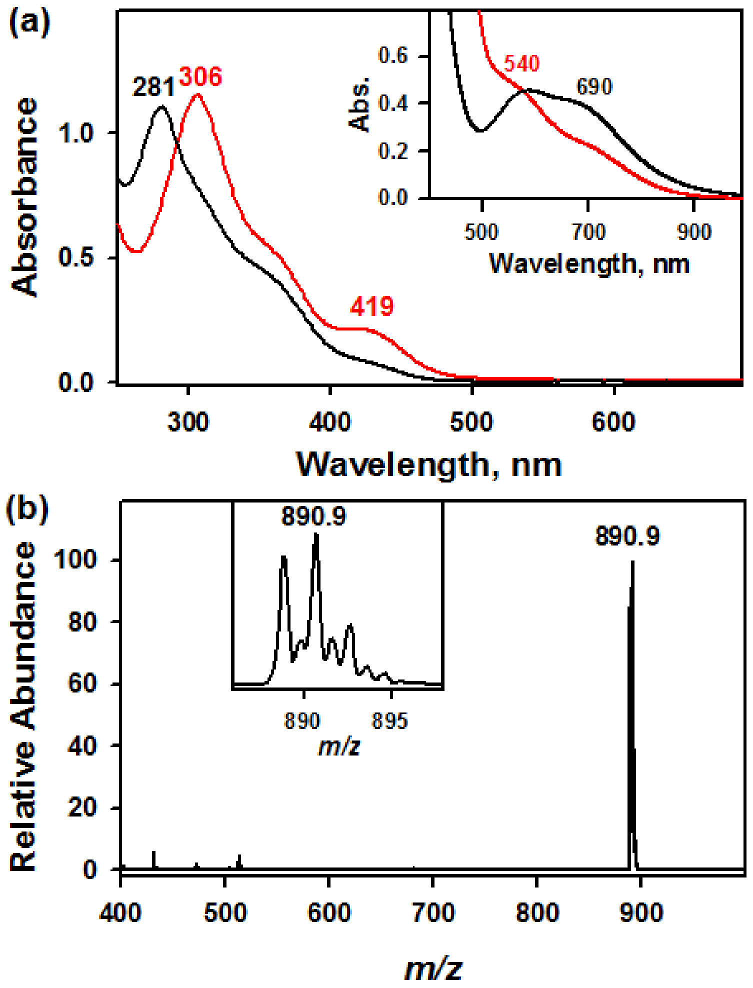

Figure S1. (a) Overalyed UV-vis spectra of **1a** (black line, 0.020 mM) and **1b** (red line, 0.020 mM) in CH_3CN and in CH_3OH at 20 ^oC, respectively. Inset shows UV-vis spectra of concentrated **1a** (black line, 1.0 mM) and concentrated **1b** (red line, 1.0 mM). (b) ESI MS spectrum of **1a** (0.020 mM) obtained in CH_3CN at 20 ^oC. A prominent peak at *m/z* of 890.9, whose mass and isotopic distribution pattern correspond to **1a** (calculated *m/z* of 891.0). Insets show the observed isotopic distribution patterns for **1a**.

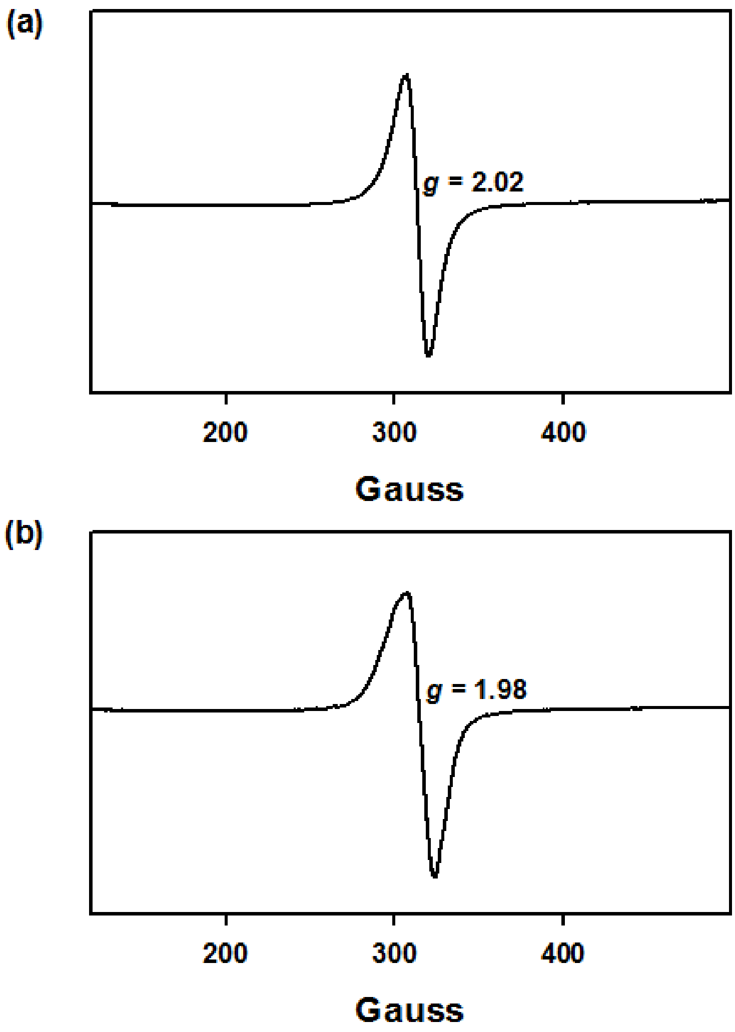

Figure S2. X-band EPR spectra of frozen solutions of (a) **1a** in CH_3CN and (b) **1b** in CH_3OH recorded at 77 K.

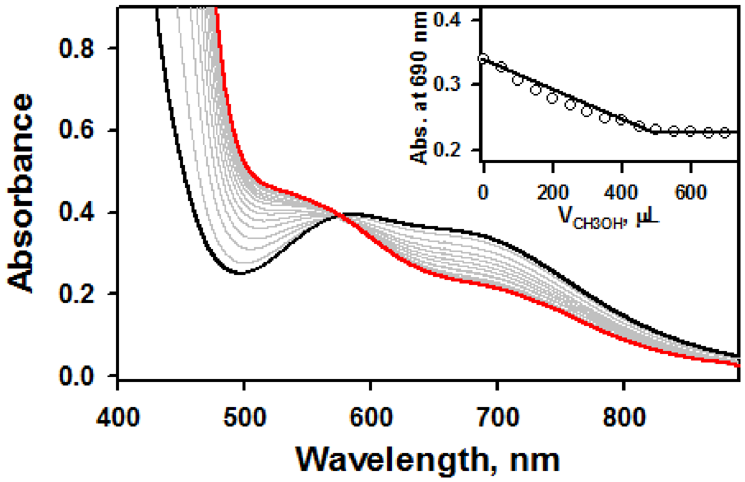

Figure S3. UV-vis spectral changes showing the formation of **1b** (red line) and disappearance of **1a** (1.0 mM, black line) upon addition of CH_3OH to **1a** in increment of 50 µL of CH_3OH at 20 °C. Inset shows the plot of absorbance changes at 690 nm due to **1a** (black dot) and against the volume of CH_3OH added to **1a** in CH_3CN at 20 °C.

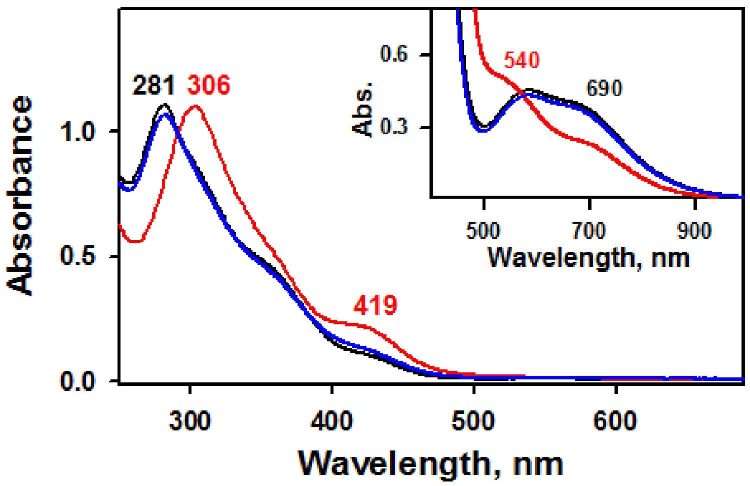

Figure S4. UV-vis spectra of **1a** (black line) and **1b** (red line) obtained in the reaction of **1a** (0.020 mM) and 2.2 equiv of KOH at 20 °C in CH_3CN. Interconversion between **1a** and **1b** was achieved by further addition of 3.0 equiv of $HClO_4$ (blue line) into the solution of **1b**.

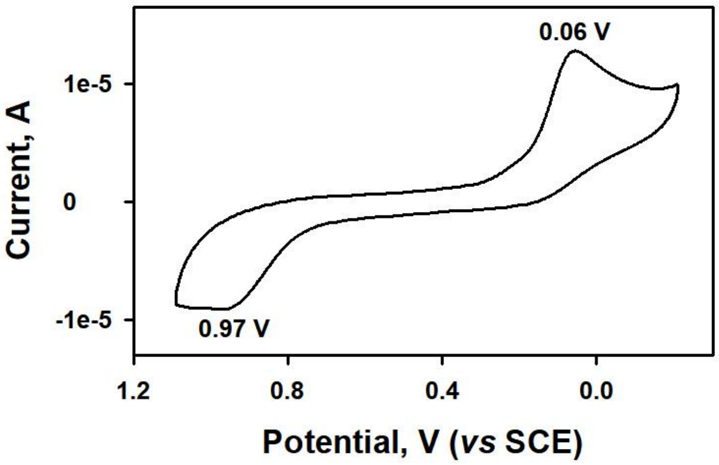

Figure S5. Cyclic voltammogram of **1a** (1.0 mM) in the presence of $TBAPF_6$ (0.10 M) in CH_3CN at 20 °C. A glassy carbon electrode was used as a working electrode. Scan was started to positive direction with a scan rate of 0.10 V s^{-1}. E_{red} of **1a** was determined to be 0.52 V vs SCE. All potentials (vs. Ag/Ag^+) were converted to values versus SCE by adding 0.29 V.

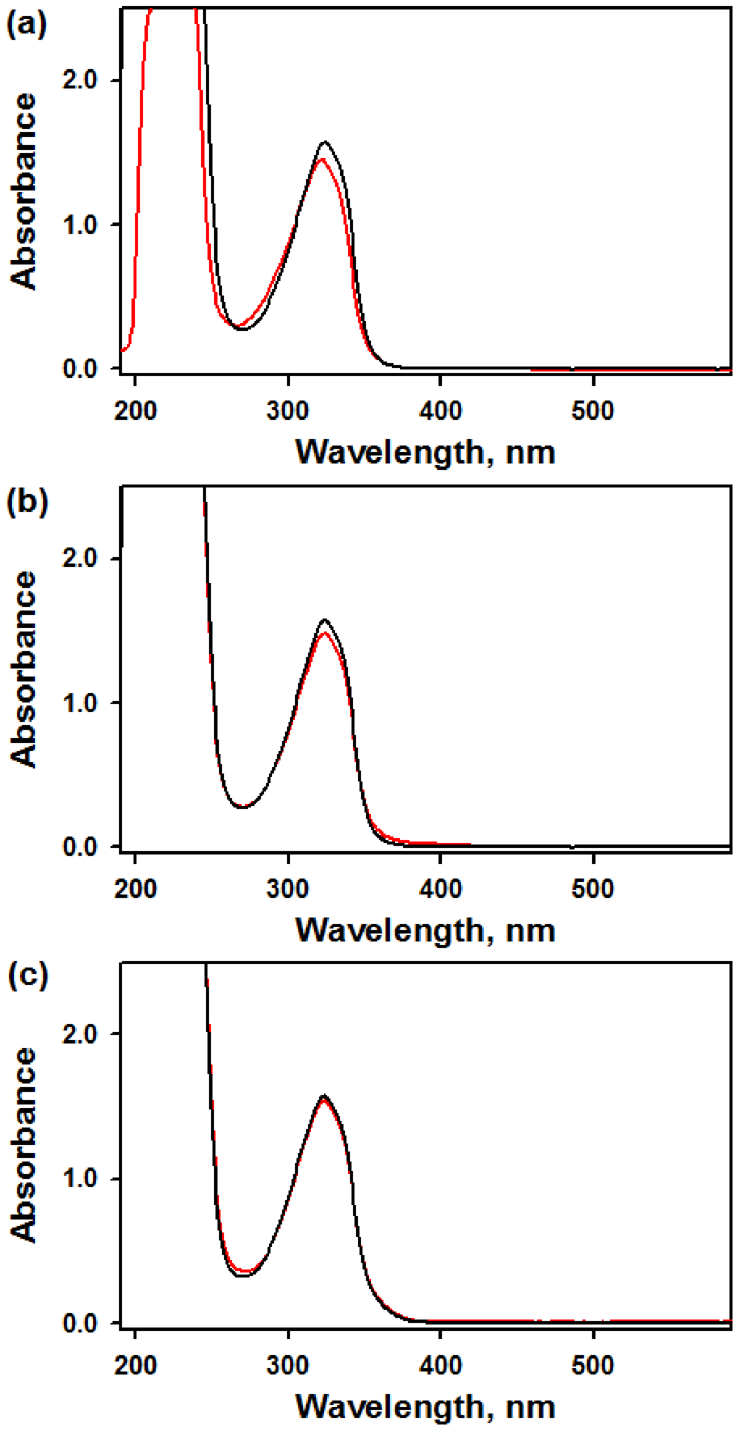

Figure S6. (a) Overlayed UV-vis spectra of bTSC ligand (0.025 mM) in CH_3CN (black line) and CH_3OH (red line) at 20 °C, respectively. (b) UV-vis spectra obtained in the reaction of bTSC ligand (0.025 mM) with 2.2 equiv of KOH in CH_3CN at 20 °C. (c) UV-vis spectra obtained in the reaction of bTSC ligand (0.025 mM) with 2.0 equiv of ferrocene in CH_3CN at 20 °C.

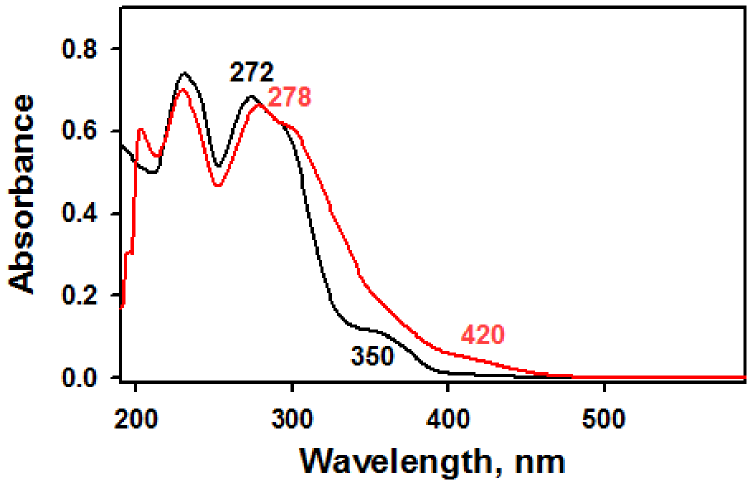

Figure S7. Overlayed UV-vis spectra of **2a** (0.025 mM) and **2b** (0.025 mM) in CH_3CN (black line) and CH_3OH (red line) at 20 °C, respectively.

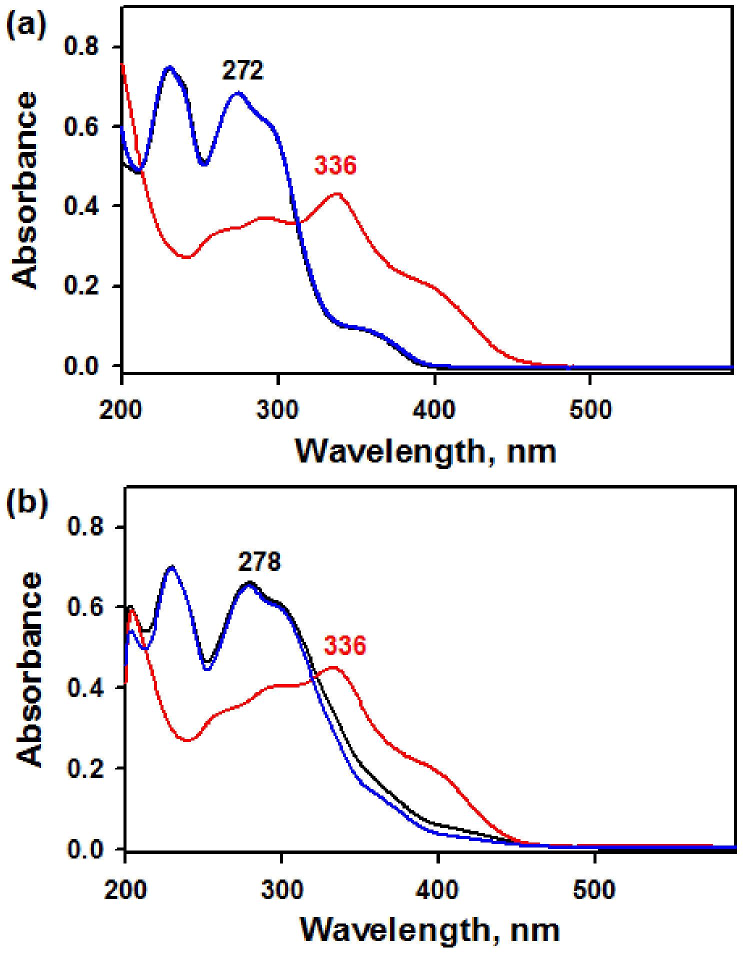

Figure S8. UV-vis spectra of **3** (red line) obtained in the reaction of (a) **2a** (black line, 0.025 mM) in CH_3CN or (b) **2b** (black line, 0.025 mM) in CH_3OH and 2.2 equiv of KOH at 20 °C, respectively. Further addition of 3.0 equiv of $HClO_4$ into the reaction solution of **3** afforded the formation of (a) **2a** (blue line) in CH_3CN and (b) **2b** (blue line) in CH_3OH at 20 °C, respectively.

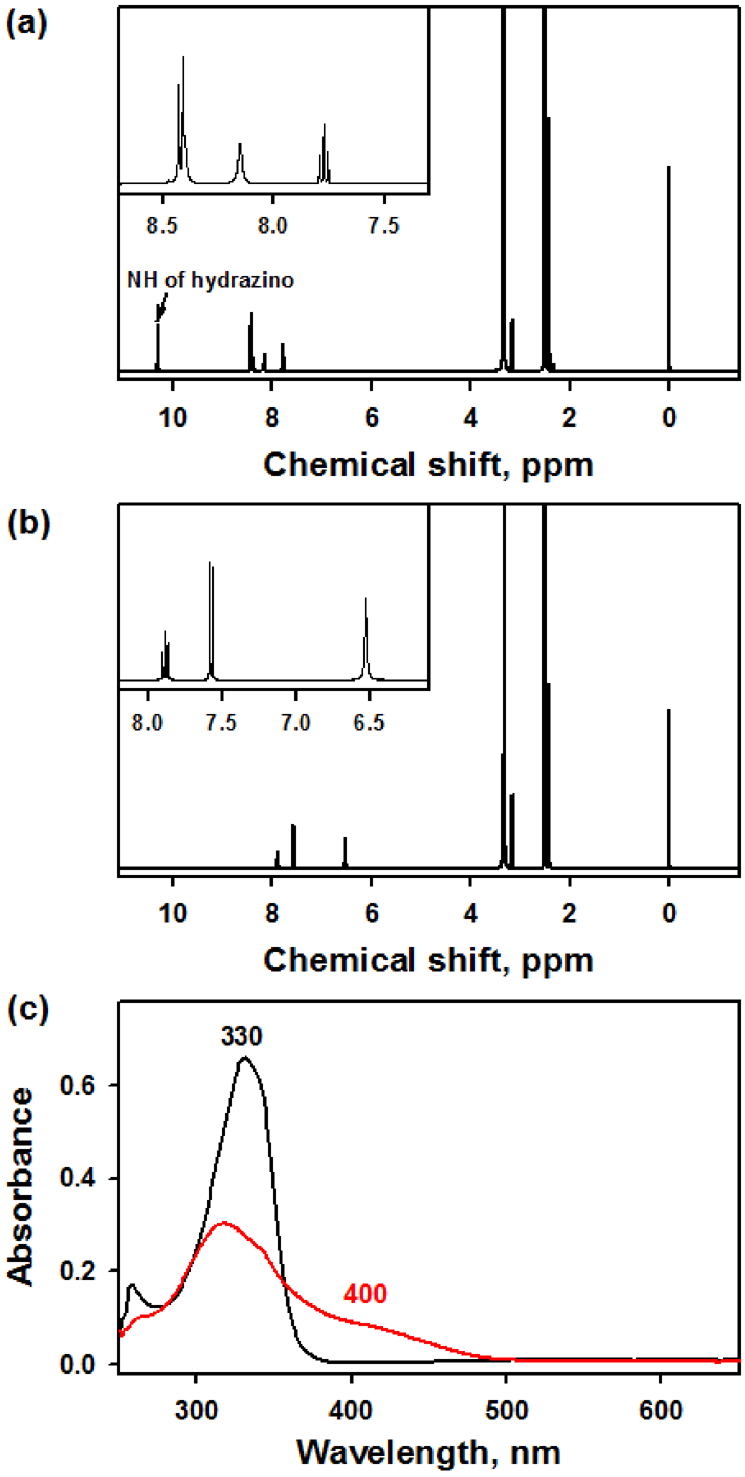

Figure S9. ^{1}H NMR spectra of (a) **2a** (4.0 mM) and (b) **3** (4.0 mM) in DMSO at 25 °C. Inset shows ^{1}H NMR spectra in the range of 6 – 11 ppm. ^{1}H NMR spectra of diamagnetic **2a** and **3** were taken at 20 °C in deutrated DMSO. (c) Overlayed spectra of **2a** (0.010 mM) and **3** (0.010 mM) dissoved in DMSO.

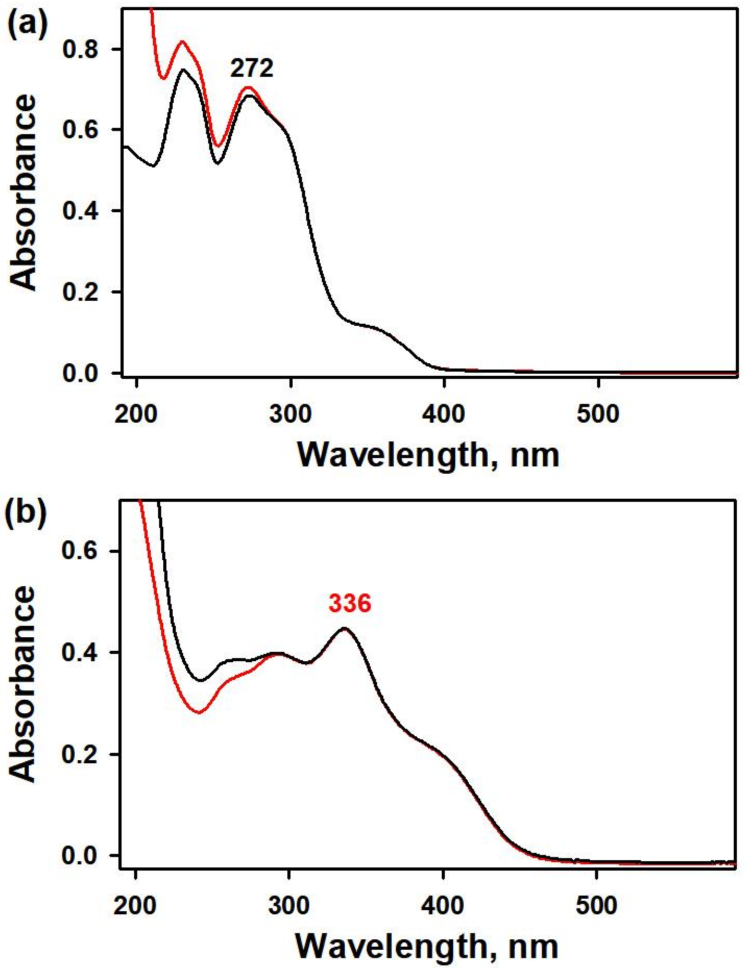

Figure S10. UV-vis spectra obtained in the reaction of (a) **2a** (0.025 mM) or (b) **3** (0.013 mM) with 1.0 equiv of ferrocene in CH_3CN at 20 ^oC.
